# Résumés of the Various Addresses

**Published:** 1906-10-06

**Authors:** 


### RÉSUMÉS OF THE VARIOUS ADDRESSES.

### THE MIDDLESEX.

#### Hospitals and the Public.

At the Middlesex Hospital an address was given by Dr. H. Campbell Thomson, M.D., F.R.C.P., and the prizes were distributed by the Lord Mayor of London. He pointed out that the position of a hospital was generally determined by the needs of a locality, but the benefits which can accrue from an institution of this kind had no such territorial limitations. Through education and research it became possible to disseminate throughout the whole world the knowledge gained in the causation and treatment of disease. It was not an easy matter for a hospital to carry out with fairness even its first duty of relieving the sick poor, for, if the definition of poverty were made too generous, the efforts of charity in one direction were marred by the creation of injustice in another. Unfortunately the use of hospitals by those who have not the proper qualifications continued to inflict a wrong on those who give and those who have a right to receive. By this abuse of charity the already depleted funds were unfairly drained; the members of the medical profession were defrauded of what they had every right to earn, and the recipient of the charity learnt to take a false view of the object of hospitals and the position of the medical profession in relation to them. It was no wonder if, in these circumstances, practitioners had come to look askance upon hospitals, and to regard their extension with alarm, instead of welcoming it as a helpful ally in difficult cases and as a means of bringing relief to the genuine poor with whom the whole medical profession will ever continue to be in sympathy. It was to be hoped that the committees of the great central funds would, by stamping their approval on well managed institutions, gradually lead the public to realise that quality is as important as quantity, and that the number of patients is by itself no guarantee of good work. And nowhere was this recognition more needful than in the out-patient departments where the methods employed are necessarily from the pressure of work too often monotonously mechanical. The most promising solution of this great out-patient problem seemed to be in the correlation of all the different forms of medical relief for the poor. It was encouraging to know that such a movement is now on foot by which it is proposed to bring about an affiliation between the hospitals and the provident dispensaries. On the subject of medical education, the lecturer observed that the question as to whether the hospitals are the proper bodies to subsidise the schools and the conditions under which this should be done was a matter which had been brought before the public so recently that he need not refer to it in detail. The essential fact, however, which it was necessary to realise was that medical education of such a standard as the public of to-day rightly demands had ceased to be a remunerative undertaking. The only way to place medical education in London on a sound basis was by endowments such as have already been forthcoming in many provincial centres, and in their own case a medical school fund, headed by the munificent gift of £1,000 from Mr. Henry Morris, had already been created.

### ST. MARY'S.

#### Science and Research.

At St. Mary's Hospital an introductory address on "The Theory and Practice of Medical Education" was delivered by Dr. N. H. Alcock, M.D., Lecturer on Physiology and Vice-Dean of the Medical School. He had endeavoured to apply a new method in order to ascertain experimentally what part is played by science in medical practice. This consisted in collecting from different practitioners, in different parts of town and country, consecutive lists of the cases they attended; 500 cases were so collected and tabulated from general practitioners and 100 from a consultant physician, giving an idea of what medical practice actually consisted. From the comparative frequency of the different diseases it appeared that the preliminary and intermediate sciences are of much use indirectly, but the actual facts are seldom required—that is, it is not the actual facts of, for example, biology that are used, but the scientific methods. The general opinion amongst the members of the medical profession who were asked was, further, that the curriculum should not be shortened nor the standard of science lowered. He also referred to the munificent offer by an anonymous donor, through Dr. Collingwood, of £100 per annum to pay for a research scholar and £100 per annum for the expenses of the research for a space of two years, and expressed a hope that other donors would be stimulated by this good example to do likewise.

### ST. GEORGE'S.

### The Qualities of a Doctor.

The Bishop of Bristol distributed the prizes and delivered an address to the students of the St. George's Hospital Medical School. Mr. C. T. Dent, surgeon to the hospital, represented the council, and there was a large attendance of students and their friends.

The Bishop of Bristol congratulated the students on being now face to face with their professional preparation for a career which each year became more scientific and therefore more interesting. There were two capacities in which he would say a few words to them. One was that of a patient. If he were asked to say what characteristic he valued most in medical advisers he would feel divided between two. The one was "cheerful gravity," the other was "grave cheerfulness." The body of man was too much under the influence—exercised for the most part unconsciously—of the will, and the unconscious will was so responsive to subtle influences that the unconscious power of a man, earnestly cheerful and earnestly grave, was a power that gave potency to the material remedies which he administered. Again, in the less acute stages of our imperfect health, the visits of a man of wide interests and wide knowledge, a man who could speak thoughtfully on many subjects, was worth very much more to the patient than the quinine or the *nux vomica*. And, for full efficacy, there must be in reserve a high moral tone. They would make a very great mistake indeed in the exercise of their profession if they thought that mere materialism afforded a full explanation of all the phenomena with which they had to deal. Their science and his science did not come from one and the same source and then diverge, but moved on absolutely parallel lines. He found no proof of his spiritual side of anything from their material side; and their material side could find no proof of anything from his spiritual side. Therefore, he was never satisfied with those who said at every wonderful discovery of science, "Here is proof of the spiritual side of things." But then, on the other hand, his position was this—each side illuminated the other in the most wonderful way; and he, moving entirely on the spiritual line, was delighted beyond measure by each discovery in physics and chemistry, because in that he saw something which not only strengthened his own faith, but gave vividness to the way his own faith spoke to him, of which he had not been aware before.

### ROYAL FREE HOSPITAL.

### Medical Women and Public Health Questions.

An introductory address was delivered to the students and graduates of the London (Royal Free Hospital) School of Medicine for Women (University of London) by Professor Byers, M.A., M.D., Belfast. Professor Byers referred to the early struggles of women doctors, and spoke of the remarkable progress of the school and of the tradition that had been created in connection with it, which it was the duty of all the students not merely to maintain, but also to intensify. Speaking first to the students, he urged upon them the importance of observation, and of doing their work systematically and in the regular order—year by year—as arranged by their teachers. Their school aimed at making the best of all that was in each of the students, and at cultivating in them the judicial faculty and the power of analysis, and at developing character—so that, apart from the necessary technical or professional education, their school endeavoured to maintain among its students the highest ideals of life and conduct, and to create among them a sense of trusteeship which would arouse a high public sentiment that it was their duty to try to make the world a little better than they found it, and to work for the betterment of human beings who surrounded them. He pointed out that there was work ready for women with education and with trained powers. There were many pressing public health questions awaiting solution in which he believed medical women would find ample scope for work:—(1) Medical inspection and supervision of school children; and (2) infantile mortality. (1) Medical inspection and supervision of school children: It was to Scandinavia, Denmark, and Germany they owed so much information in reference to the health of school children, and he drew attention to the recent step taken in Philadelphia where a daily clinique has been started at which the elementary school children are to be examined as to both their bodily and mental condition, and where suitable treatment, including operations when required, will be recommended (say for shortsightedness, throat deafness, etc.). Surely in such important work as school inspection, there were ample openings for medical women.

### UNIVERSITY COLLEGE, LONDON.

### Then and Now.

The inaugural address at the School of Advanced Medical Studies, University College, was delivered by Professor Rickman J. Godlee.

Mr. Godlee compared the educational difficulties of the 1826 period with those of the present day. The object of the University was said to be "the advancement and promotion of literature and science by affording to young men residing in or resorting to the cities of London and Westminster, the borough of Southwark, and the counties adjoining, adequate opportunities of obtaining literary and scientific education at a moderate expense." The scheme raised great opposition from the Churchmen and Tories, but it rapidly proved to be a great success. The part first opened was the Faculty of Medicine, which immediately attracted large numbers of students. There was no hospital attached to the University, but Charles (afterwards Sir Charles) Bell and Dr. (afterwards Sir Thomas) Watson, who were on the staff of Middlesex Hospital, were professors at the University, and the students of the University were allowed special facilities for obtaining clinical instruction at the Middlesex Hospital. A dispensary was started at No. 4 George Street in 1828. It was called the "University Dispensary," and was under the *ægis* of the University. The "North London" Hospital was founded in 1833, and was completed in 1846. In 1837 its name was changed to "University College Hospital," and in 1851 to "North London or University College Hospital," by which it is known at the present day. The lecturer then described in some detail the precise position of the new corporation, its capacity, equipment, and objects, and stated his views as to what its prospects and functions should be.

### KING'S COLLEGE.

### Methods: The Old and the New.

Mr. T. Pridgin Teale, consulting surgeon to the General Infirmary at Leeds, gave an introductory address at King's College Hospital under the title of "A Retrospect."

Mr. Teale said that he was one of a very small number now living who had seen through and taken part in the greatest revolution in surgical and medical practice and in medical education that probably the world had ever known, and one of a still smaller band of men whose student life began in the original King's College Hospital. The very name, King's College Hospital, was an epitome of its history. When the medical school of King's College was first established it had no hospital of its own, its students being dependent upon the hospitality of an alien hospital, not conveniently situated for students working at the college in the Strand. Feeling the need of a hospital of their own, at a more convenient distance from the school and under the control of the staff, the authorities purchased the old Clement Danes Workhouse, flanked by a disused burial-ground as an "open space." Such was the hospital to which he came as a student in 1852. The theatre served the double purpose of providing for operations and for post-mortem examinations, the dead-house being placed beneath a trap-door in the floor of the operating theatre. It was during his studentship that the present hospital was commenced, and the first portion was completed and brought into use in his final year, the initial year of the Crimean War. The old workhouse hospital served its purpose in its day, was the scene of the advanced surgery of its time, and was the sphere of work of one of England's greatest clinical teachers, Todd, and of one of our greatest surgeons, Fergusson. Its cost was probably under £100,000, and it had probably 100 beds. The new hospital would be planned in the first instance for 408 beds, with a possible increase to 600, and the preliminary estimate of cost is £366,712. What a contrast! And what a lesson was to be learned from its consideration!

### THE LEEDS UNIVERSITY.

### English Neglect of Science.

The inaugural address was delivered by Sir James Crichton Browne on "Universities and Medical Education."

Sir James was not at all sure that this age of science, with its colossal wealth, had done more for the endowment of learning than did the ages of faith in their chill penury. The remembrance of the zeal for learning of our ancestors, of the generosity and sagacious foresight with which they fostered it, should rouse us to redoubled effort and incite us to take long views of the future. Much had been done, but much remained to do. No University could write the word "limited" after its name, least of all a University like that of Leeds, which is responsible for adapting its teaching and research in some measure to the ever-growing and changing needs of a great industrial community. We had quite recently, at the celebration of the 50th anniversary of the discovery of the first dye stuff extracted from coal tar by Sir William Perkin, had our attention forcibly drawn to our loss of an industry that rightly belonged to us, owing to our lack of technical knowledge and insight. The basic discoveries in connection with coal-tar industries, as Sir James Dewar had pointed out, were made in England; we had the raw material, we had the capital, we had the skilled labour, but we allowed the profitable trade in dyes, perfumes, and drugs, which is now said to be worth 50 millions per annum, to pass to Germany, simply because we had not at the time the educational machinery to carry out the experimental research necessary to enable us to take advantage of our own discoveries. England had been remiss of late in perceiving and promoting those interests that hinge on scientific and medical research. In this direction Germany had stolen a march upon us, for the various Governments in that empire had unstintedly provided their Universities with fully-equipped research laboratories, organised and conducted by professorial directors. The public neglect of medical education in this country or indifference towards it was really, when we came to think of it, astonishing. The public concernment in it was direct and immediate. Few men or women entered life or got through it or left it without medical assistance. In no other of the great professions had less been done by the State aid or private philanthropy to supplement the payments of those seeking to enter it. It was in research work in medicine that Germany is pushing ahead, and in which we must endeavour to overtake her; and that, if given the chance, we shall overtake her could scarcely be questioned, for with all the advantages she has enjoyed up till now we have no cause to shrink from comparison with her in medical science.

